# Vacuolar-ATPase-mediated muscle acidification caused muscular mechanical nociceptive hypersensitivity after chronic stress in rats, which involved extracellular matrix proteoglycan and ASIC3

**DOI:** 10.1038/s41598-023-39633-1

**Published:** 2023-08-21

**Authors:** Teruaki Nasu, Amane Hori, Norio Hotta, Chiaki Kihara, Asako Kubo, Kimiaki Katanosaka, Masamitsu Suzuki, Kazue Mizumura

**Affiliations:** 1 Department of Biomedical Sciences, College of Life and Health Sciences, Chubu University, Matsumoto-Cho, Kasugai, 487-8501 Japan; 2 Graduate School of Life and Health Sciences, Chubu University, Matsumoto-Cho, Kasugai, 487-8501 Japan; 3 Japan Society for the Promotion of Science, Kojimachi, Chiyoda-Ku, Tokyo, 102-8472 Japan; 4 Department of Lifelong Sports and Health Sciences, College of Life and Health Sciences, Chubu University, Matsumoto-Cho, Kasugai, 487-8501 Japan; 5 Department of Physiology, Nihon University School of Dentistry, 1-8-13 Kandasurugadai, Chiyoda-Ku, Tokyo, 101-8310 Japan; 6 Present Address: Department of Acupuncture and Moxibustion, Faculty of Rehabilitation, Niigata University of Health and Welfare, Niigata, 950-3198 Japan; 7 Central Research Laboratories, ZERIA Pharmaceutical Co. Ltd., 2512-1 Numagami, Oshikiri, Kumagaya, Saitama, 360-0111 Japan; 8 Department of Physical Therapy, College of Life and Health Sciences, Chubu University, Matsumoto-Cho, Kasugai, 487-8501 Japan

**Keywords:** Neuroscience, Physiology, Medical research

## Abstract

Although widespread pain, such as fibromyalgia, is considered to have a central cause, peripheral input is important. We used a rat repeated cold stress (RCS) model with many characteristics common to fibromyalgia and studied the possible involvement of decreased muscle pH in muscle mechanical hyperalgesia. After a 5-day RCS, the muscle pH and the muscular mechanical withdrawal threshold (MMWT) decreased significantly. Subcutaneously injected specific inhibitor of vacuolar ATPase (V-ATPase), bafilomycin A1, reversed both changes almost completely. It also reversed the increased mechanical response of muscle thin-fibre afferents after RCS. These results show that V-ATPase activation caused muscle pH drop, which led to mechanical hypersensitivity after RCS. Since extracellular matrix proteoglycan and acid sensitive ion channels (TRPV1 and ASIC3) have been considered as possible mechanisms for sensitizing/activating nociceptors by protons, we investigated their involvement. Manipulating the extracellular matrix proteoglycan with chondroitin sulfate and chondroitinase ABC reversed the MMWT decrease after RCS, supporting the involvement of the extracellular mechanism. Inhibiting ASIC3, but not TRPV1, reversed the decreased MMWT after RCS, and ASIC3 mRNA and protein in the dorsal root ganglia were upregulated, indicating ASIC3 involvement. These findings suggest that extracellular mechanism and ASIC3 play essential roles in proton-induced mechanical hyperalgesia after RCS.

## Introduction

Fibromyalgia is characterised by chronic widespread pain, especially in deep tissues such as muscles; however, it is accompanied by multiple symptoms, including psychological distress, autonomic dysfunction, sleep disturbances, fatigue, and other functional disorders^1^. It is classified as a nociplastic pain^2^ and is considered as having a central cause^1^. In addition, the involvement of the peripheral mechanism has been noted in its maintenance^3,4^; however, the factors that induce nociceptor activation/sensitization are unclear. Many animal models have been developed to study the pathophysiological mechanism and pharmacological study of fibromyalgia and repeated cold stress/ intermittent cold stress models (RCS/ICS), in which animals were repeatedly (intermittently) exposed to 4–6 °C and 22–24 °C every 30 min, have symptomatic similarities with fibromyalgia, such as generalized hyperalgesia, especially in deep tissues lasting up to 3 weeks, psychological distress (depression and anxiety), and autonomic dysfunctions^5–10^. The involvement of inflammation has been proposed in an ICS mouse model^11^. However, we could not detect altered expression of cytokines and neurotrophic factors in rat muscles in the RCS model^12^.

Another possible cause of muscular pain is acidic conditions. Acidic conditions often occur in the muscles during inflammation^13,14^, ischaemic contraction^15^, myofascial trigger points^16^, and exhausting exercise^17^. Protons induce a nociceptive sensation in the skin and muscle^15,18^ and decrease the response threshold of nociceptors to mechanical stimulation^19^, thereby contributing to mechanical hypersensitivity. In addition, Sluka et al.^20^ reported that injection of an acidic solution into the muscle twice, with an interval of 5 days, induces long-lasting bilateral mechanical hyperalgesia.

Tissue acidification can be induced by the activation of vacuolar ATPase (V-ATPase). V-ATPase is an ATP-dependent proton pump originally found in the intracellular compartment and it comprises 14 subunits and transports protons to intracellular organelles^21^. Some cells express V-ATPase on the plasma membrane where they perform cell-specific functions such as renal acidification^22^, bone resorption^23^, and neuronal transmission^24^. An important role in tumour metastasis has also been proposed^25^. However, the involvement of V-ATPase in painful muscular conditions has not been studied.

Ion channels that sense low pH are transient receptor potential vanilloid 1 (TRPV1) and acid-sensing ion channel 3 (ASIC3), which have different pH ranges for activation^26,27^. The involvement of these channels in the sensitisation of nociceptive afferents in mechanical nociceptive hypersensitivity has been reported in inflammation^28–30^ and nerve injury^31^; however, it is unclear whether low pH is involved in the sensitising mechanism through activation of these channels. We found another mechanism by which protons sensitise nociceptors to mechanical stimulation that does not involve TRPV1 or ASICs: Extracellular matrix proteoglycans are crucial in this sensitisation; however, proton-induced inward currents except rapid adapting type, are not inhibited by a combination of a selective TRPV1 antagonist and an ASICs antagonist^32^. This mechanism was first found in the current response to mechanical indentation of cultured dorsal root ganglion (DRG) neurones^32^. It was then confirmed at the tissue level by recording single thin-fibre activities from the muscle^33^. Although no involvement of ASICs and TRPV1 was implicated in proton-induced sensitization to mechanical stimulation in normal cultured DRG neurones^32^, it might be changed in pathological condition such as RCS. Therefore, we studied involvement of all three mechanisms (extracellular mechanism, TRPV1 and ASICs) in proton-induced mechanical nociceptive hypersensitivity in this study.

Herein, we show that muscle pH is reduced by activating V-ATPase after RCS^9,34^, resulting in muscle nociceptive hypersensitivity to mechanical stimulation. Furthermore, we show that this mechanical hypersensitivity is mediated via the extracellular matrix proteoglycan, a mechanism previously proposed by Kubo et al*.*^32^, and additionally by ASIC3.

Preliminary results of the present study were presented in abstract form^35–37^.

## Results

### Change in muscle pH after RCS

**Figure 1:**
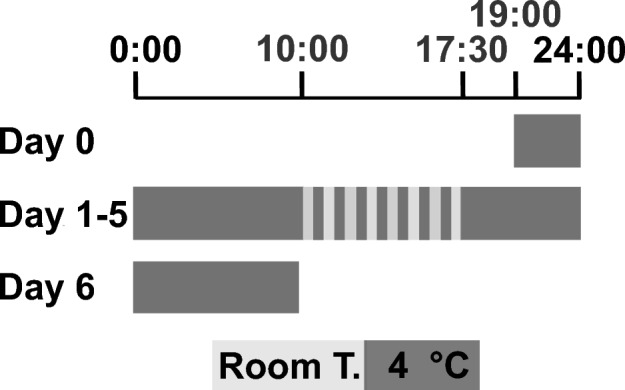
Schedule for repeated cold stress (RCS) application. The dark columns show the periods when animals were placed in a room at low temperature (4 °C), and light gray columns show those at room temperature (22–24 °C).

RCS was applied, as shown in Fig. 1.

**Figure 2:**
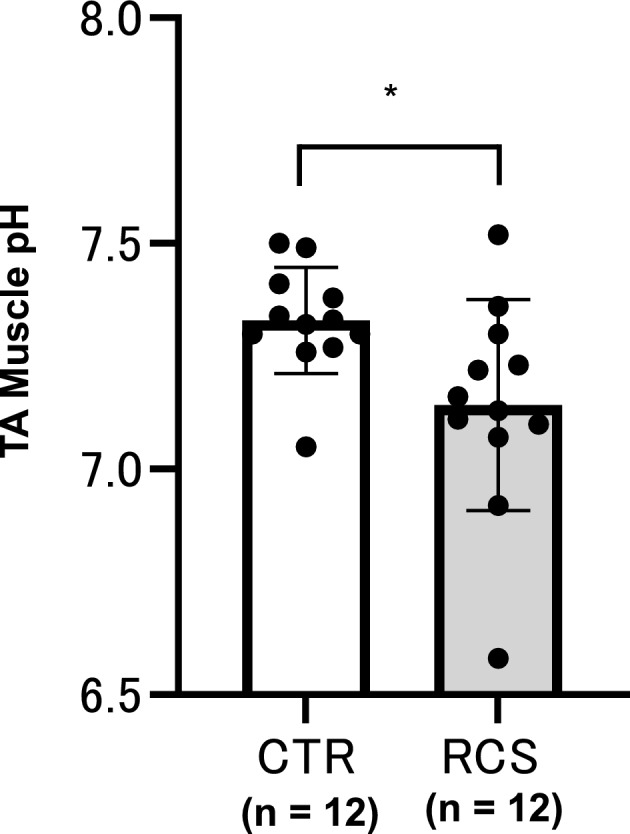
Change in muscle pH after RCS. The pH of the TA muscle was measured. CTR, control group; RCS, RCS group (3–5 days after RCS). Bar graphs are shown (mean ± SD). Black dots indicate each data point. Unpaired t-test with Welch’s correction detected a significant difference (*: *p* = 0.0245) between the control and RCS groups. Statistical summary in Supplementary Table 1.

Since the pH of the tibialis anterior (TA) muscle in normal animals under isoflurane anaesthesia was 7.33 ± 0.12 (n = 12), and that in animals 3–5 days after RCS was 7.14 ± 0.23 (n = 12), the pH of the RCS group was significantly lower than that of the control (unpaired t-test with Welch’s correction, *p *= 0.0245, Supplementary Table 1) (Fig. 2).

### Effect of a V-ATPase inhibitor, bafilomycin A1, on the decreased muscular withdrawal threshold after RCS

**Figure 3:**
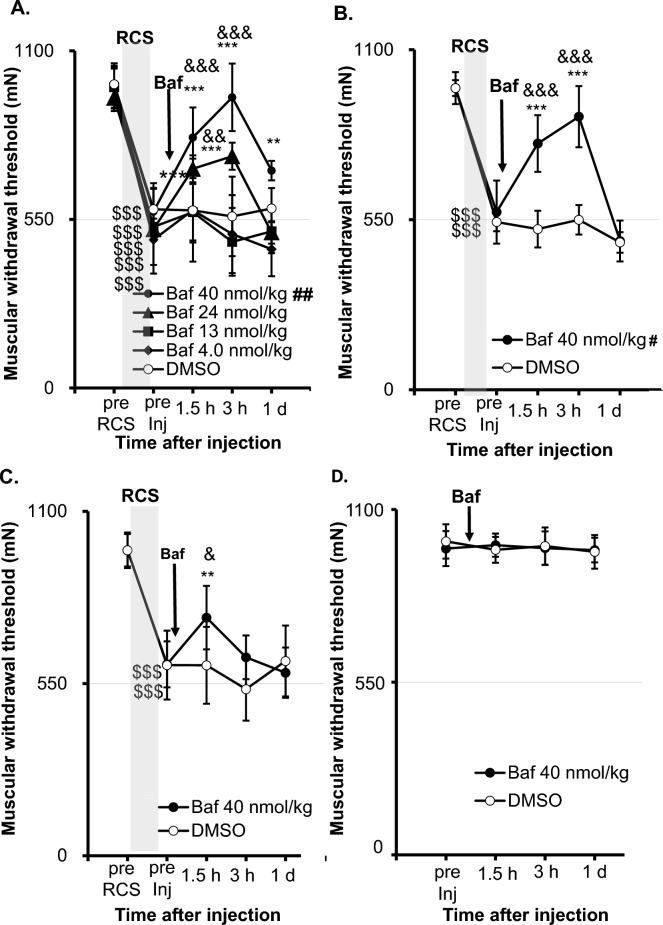
Effects of bafilomycin A1, a specific inhibitor of vacuolar ATPase (V-ATPase), on the decreased withdrawal threshold at various periods after RCS. Vertical axis: muscular mechanical withdrawal threshold (MMWT) in mN; horizontal axis: time (non-linear). (**A**) One week after the RCS. (**B**)Two weeks after the RCS. (**C**) Three weeks after the RCS. (**D**) Normal control without RCS exposure. The vertical gray column indicates the period of the RCS, “Baf” with an arrow indicating the time when bafilomycin A1 was injected. Two-way repeated measures ANOVA (see Supplementary Table 2 for statistics summary) was followed by post hoc analyses using Bonferroni’s test. #, ##: *p* < 0.05, 0.01 compared to the DMSO group (main effect of “drug”). $$$: *p* < 0.001 compared to “pre RCS”, **, ***: *p* < 0.01, 0.001 compared to “pre Inj”, &, &&, &&&: *p* < 0.05, 0.01, 0.001 compared to the DMSO group at corresponding time point. MMWT decreased after RCS in all dose groups. Bafilomycin A1 24 and/or 40 nmol/kg groups showed significantly higher MMWT than the DMSO group at 1 week (*p* < 0.01) and 2 weeks (*p* < 0.05) after RCS. A significant reversal of the decrease in MMWT was detected at 1.5 h. It lasted up to 1 day after injection with 40 nmol/kg bafilomycin A1, and a significant reversal was observed 1.5 h after injection, which disappeared 1 day after injection with 24 nmol/kg (**A**). Bafilomycin A1 40 nmol/kg also reversed the decreased MMWT up to 3 h after its injection 2 weeks after RCS (**B**). This effect is weak and lasted only up to 1.5 h after bafilomycin A1 injection 3 weeks after RCS (**C**). Bafilomycin A1 40 nmol/kg did not affect the control animals without RCS (**D**).

To determine whether the decreased pH of the muscle after RCS plays any role in muscular nociceptive hypersensitivity, we examined the effects of bafilomycin A1, a specific inhibitor of V-ATPase^38^ on the decreased muscular mechanical withdrawal threshold (MMWT) at different time points after RCS (Fig. 3). Statistical analysis with two-way repeated measures ANOVA showed significant effects in [time], [drug], and [time] × [drug] interactions (n = 6 each, see Supplementary Table 2) at 1 week (Fig. 3A). Post-hoc analysis with Bonferroni’s test showed that there was a significant difference in MMWT between before RCS (“pre RCS”) and before injection of bafilomycin A1 (“pre Inj”) in all groups with different doses of bafilomycin A1 and dimethyl sulfoxide (DMSO, vehicle) 1 week after RCS, i.e., MMWT decreased after RCS (indicated as “pre Inj”. *p* < 0.001, for all dose groups), confirming our previous data^9,39^. This muscular mechanical hypersensitivity after RCS was partially reversed by bafilomycin A1 at 24 nmol/kg (*p* < 0.001 for 1.5 and 3 h after injection vs. before injection (“pre Inj”)) and almost completely reversed by bafilomycin A1 40 nmol/kg (*p* < 0.001 for 1.5 and 3 h after injection and p < 0.008 for “1 d” vs “pre Inj”). Doses of 4.0 and 13 nmol/kg had no significant effect. Group comparison showed a significant difference between the DMSO group and that treated with only bafilomycin A1 40 nmol/kg (*p* = 0.01). MMWT was significantly higher than that of the DMSO group 3 h after bafilomycin A1 24 nmol/kg (*p* < 0.005) and 1.5 h and 3 h after bafilomycin A1 40 nmol/kg injection (*p* < 0.001 for both time points). Vehicle (DMSO) injection did not change MMWT (*p* = 1 at all time points). This dose–response relation is clearly seen in AUC graph (Supplementary Fig. S1A). Since bafilomycin A1 40 nmol/kg completely reversed MMWT 1 week after RCS, we used only this dose for subsequent experiments (Fig. 3B–D).

Two weeks after RCS the decreased withdrawal threshold after RCS was reversed by bafilomycin A1 40 nmol/kg up to 3 h after the injection (*p* < 0.001) and became even a little lower than “pre Inj” 1 d after injection (*p* = 0.324, Fig. 3B). MMWT in the bafilomycin A1 group was significantly higher than that in the DMSO group 1.5 h and 3 h after injection (Fig. 3B, *p* < 0.001, also see Supplementary Fig. S1B for AUC). Three weeks after RCS, the effect of bafilomycin A1 was shorter, i.e., MMWT was significantly higher than after DMSO (*p* = 0.011) and also higher than that of “pre Inj” (*p* = 0.002) only at time point 1.5 h after bafilomycin A1 40 nmol/kg (Fig. 3C), and no significant change in AUC (Supplementary Fig. S1C). Notably, the administration of DMSO or bafilomycin A1 induced no change in the withdrawal threshold in naïve (without RCS) rats for up to 1 d after injection (Fig. 3D, and Supplementary Fig. 1D).

**Figure 4:**
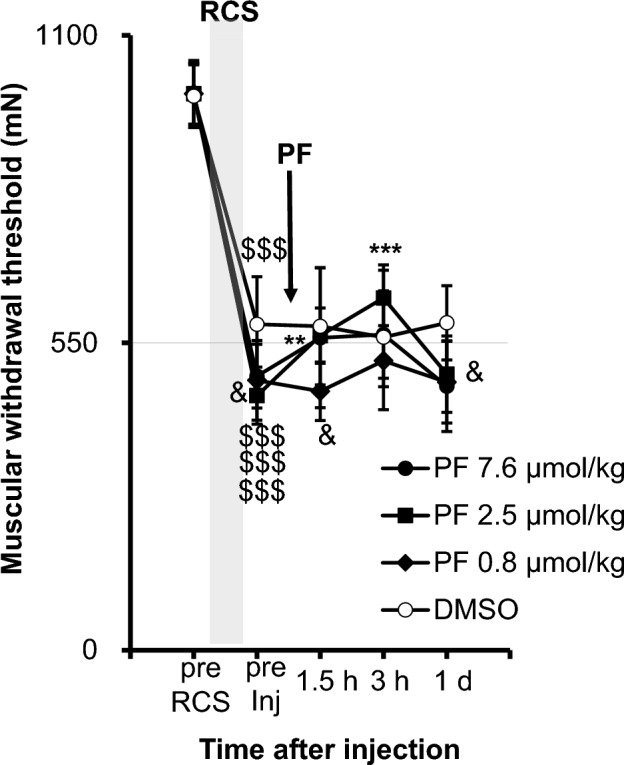
Effect of a specific inhibitor for proton pump, PF3716556, on the decrease in muscular mechanical withdrawal threshold after RCS. The presentation method was the same as that shown in Fig. 3. Two-way repeated measures ANOVA (see Supplementary Table 3 for statistics summary) was followed by post hoc analyses using Bonferroni’s test. $$$: *p* < 0.001 compared to ”pre RCS”, **, ***: *p* < 0.01, 0.001 compared to “pre Inj”, &: *p* < 0.05, compared to the DMSO group at the corresponding time point. PF3716556 (PF) with an arrow indicates the time PF was injected. This experiment was performed one week after RCS. PF 2.5 μmol/kg showed some reversal of the withdrawal threshold at 1.5 h and 3 h after injection, but this effect was small compared to that with bafilomycin A1, as shown in Fig. 3A.

We examined whether another proton pump, H^+^/K^+^-ATPase, was involved in mechanical hypersensitivity after RCS. For this, we used a specific inhibitor of H^+^/K^+^-ATPase, PF3716556, in rats 1 week after RCS. As shown in Fig. 4 (statistical summary in Supplementary Table 3), the withdrawal threshold decrease after RCS was slightly but significantly reversed with PF3716556 only at a dose of 2.5 μmol/kg (*p* < 0.01 1.5 h after injection, *p* < 0.001 3 h after injection), in comparison, 7.6 μmol/kg did not reverse the threshold. This dose-relationship is clearly seen in AUC graph (Supplementary Fig. S2). %Maximal possible effect (MPE) of PF3716556 2.5 μmol/kg 3 h after injection was 32.7 ± 6.7%. It was much smaller than that of bafilomycin A1 40 nmol/kg 3 h after injection (90.5 ± 22.7%), and the difference between these two values was statistically significant (*p* < 0.0017, n = 6 each, unpaired t-test with Welch’s correction, Supplementary Table 1).

### Change in pH after injection of bafilomycin A1

**Figure 5:**
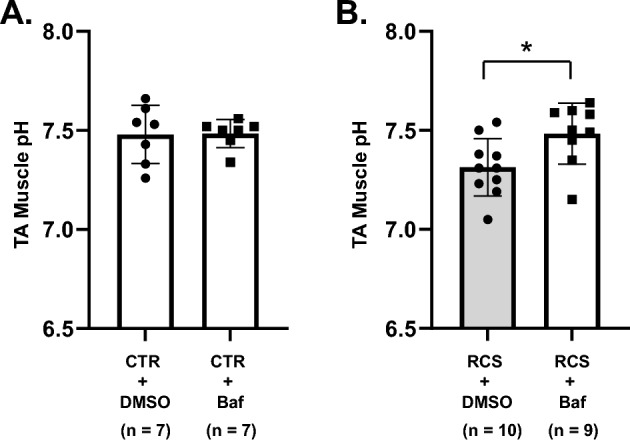
Change in muscle pH by bafilomycin A1. Bar graphs (mean ± SD) with each data point are shown. (**A**) Effects of bafilomycin A1 and vehicle DMSO on the control muscle in rats without RCS. (**B**) Effects of bafilomycin A1 and vehicle DMSO on muscles that underwent RCS (studied one week after RCS). Bafilomycin A1 significantly increased the pH of the muscle that underwent RCS (B, *: p < 0.0244, unpaired t-test with Welch’s correction), whereas it did not affect the pH of the control muscle ((**A**), *p* = 0.9461). Supplementary Table 1 for statistics summary.

Next, we measured muscle pH after bafilomycin A1 treatment one week after RCS to examine whether the effect of bafilomycin A1 on the decreased MMWT after RCS was actually due to the reversal of decreased pH. For this, we used bafilomycin A1 40 nmol/kg, the most effective and highest dose in the withdrawal threshold measurement series. The muscle pH after the injection of bafilomycin A1 (7.48 ± 0.15) was not different from that after vehicle (DMSO) injection in the control animals for the DMSO group; 7.48 ± 0.07 for the bafilomycin A1 group, n = 7 each, *p *= 0.9461, unpaired t-test with Welch’s correction, Supplementary Table 1) (Fig. 5A). When we blocked V-ATPase activity in RCS rats, the pH of the TA muscle increased significantly from pH 7.31 ± 0.15 of vehicle-injected muscle (n = 10, injected with DMSO) to 7.48 ± 0.15 (n = 9, *p* = 0.0244. unpaired t-test with Welch’s correction, Supplementary Table 1) measured within 1.5–3 h after bafilomycin A1 injection (Fig. 5B).

### Single fibre recording

**Figure 6:**
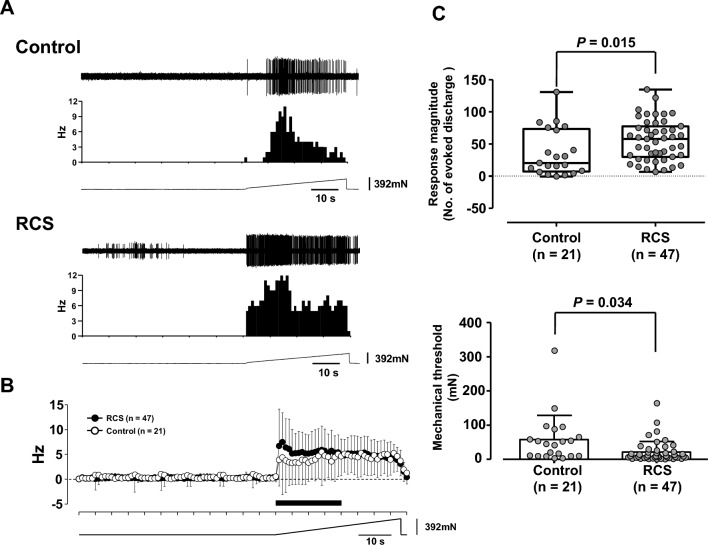
Mechanical responses of single muscle thin-fibre afferents recorded ex vivo from control and RCS rats. (**A**) Sample recordings of the mechanical responses of muscle thin-fibre afferents of control (upper column, conduction velocity 0.71 m/s) and RCS (lower column, conduction velocity 0.37 m/s, within 3 weeks after RCS) rats. From top to bottom, raw recordings of neural activities and their peri-stimulus time histograms (1 bin is 1 s, scale on the left) and raw recordings of the force output of the mechanical stimulator are presented. Scales for time and force are shown at the bottom right of each force recording. The small voltage spikes observed in the RCS specimen are due to untargeted neural activities. (**B**) Averaged peri-stimulus time histograms of mechanical responses (mean + or − SD). The black circles represent the response of RCS, and the white circles represent the control. The horizontal and vertical axes represent the time in seconds and the discharge rate in impulses/s, respectively. The black bar under the histograms indicates the period during which the response magnitude is calculated. (**C**) Comparison of the response magnitude (upper graph) and response threshold to mechanical stimulation (mechanical threshold, lower graph) between the control and RCS groups. The data are presented as boxes and whiskers (upper graph) and as mean ± SD (lower graph) with each data point. The Mann–Whitney U test (for the response magnitude) and unpaired t-test with Welch’s correction (for the response threshold) showed significant differences between RCS and the control both in the response magnitude (*p* = 0.015) and mechanical threshold (*p* = 0.034). Supplementary Table 1 for statistics summary.

To ascertain whether changes in muscle pH influence peripheral afferent nerve activity, we performed single-fibre recording of C (Group IV) fibres from the extensor digitorum longus (EDL) muscle and examined the general response characteristics and effects of bafilomycin A1. The basic characteristics of the control and RCS fibres and the change in the mechanical response by RCS were similar to those reported by Wakatsuki et al*.*^12^. In brief, there were no differences in conduction velocity and background activity between the control and RCS groups (0.8 m/s [interquartile range, IQR] 0.6–0.9 m/s] vs. 0.6 m/s [IQR 0.5–0.8 m/s] and 0.1 impulses/s [IQR 0.0–0.4 impulses/s] vs. 0.1 impulses/s [IQR 0.0–0.4 impulses/s], respectively [*p* = 0.094 and *p* = 0.746, respectively, Mann–Whitney U test], Supplementary Table 1). As shown in Fig. 6A, mechanical stimulation induced a rapid increase in the discharge rate, especially in the RCS fibre, at the beginning of the stimulus and then declined to become steady at a slightly lower discharge rate in the latter half of the stimulation applied for 38 s. This pattern was more clearly observed in the average time histogram of discharge (Fig. 6B). The difference between the control and RCS groups appeared to exist only during the early half of the stimulus period. Therefore, the responses during the first 20 s of the stimulus period (marked with a black bar in Fig. 6B) were used to analyse the response magnitude. The magnitude of the mechanical response was significantly larger in the RCS group (Fig. 6C, upper graph, *p* = 0.015, Mann–Whitney U test, Supplementary Table 1), and the threshold of the mechanical response was significantly lower in the RCS group (Fig. 6C, lower graph, *p* = 0.034, unpaired t test with Welch’s correction, Supplementary Table 1).

### Effect of bafilomycin A1 on muscle C-fibre responses to mechanical stimulation

**Figure 7:**
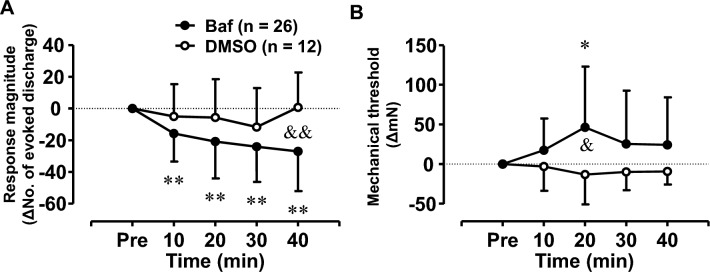
Effect of bafilomycin A1 on mechanical responses of muscle thin-fibre afferents recorded from RCS rats. Changes in the response magnitude (**A**) and mechanical threshold (**B**) of afferent fibres were recorded in RCS rats (within 3 weeks after RCS) after injection with bafilomycin A1 4 μM (Baf, black circle) or vehicle (2.5% DMSO, white circle). The vertical axis in A: the change in the mechanically evoked discharges from the “Pre” value before injection; the horizontal axis: the time after bafilomycin A1 or DMSO injection. (**A**) Two-way repeated-measures ANOVA (see Supplementary Table 3 for a statistics summary) followed by post hoc analyses using Bonferroni’s test. Post-hoc tests showed that the response magnitude after bafilomycin A1 injection decreased significantly at all time points, whereas that after DMSO injection remained unchanged. Therefore, the response magnitude of the bafilomycin group was significantly smaller than that of the DMSO group 40 min after intramuscular injection. (**B**) The mechanical threshold increased significantly in the bafilomycin group at 20 min than “Pre”, and was significantly higher than in the DMSO group. *, **: *p* < 0.05, 0.01 vs. “Pre”; &, &&: *p:* < 0.05, 0.01 vs. DMSO group.

We examined whether this increased response in the RCS group was induced by muscle acidification via V-ATPase activity. Bafilomycin A1 (4 μM-5 μL) or vehicle (2.5% DMSO-5 μL) was injected into the muscle near the receptive field of the recorded fibre, and the mechanical responses of the fibres in the bafilomycin (n = 26) and vehicle (DMSO) groups (n = 12) were recorded from rats that underwent RCS. The responses before bafilomycin A1 or vehicle injection did not differ between the two groups (response magnitude [No. of evoked discharges]: 59.6 ± 34.6 impulses for bafilomycin A1group vs. 46.8 ± 23.9 impulses for DMSO group, *p* = 0.257, unpaired Student t-test; threshold 9.4 mN [IQR 5.0–18.0 mN] for bafilomycin A1 group vs. 16.7 mN [IQR 6.3–31.5 mN] for DMSO group, *p* = 0.376, Mann–Whitney U test. Supplementary Table 1). Therefore, the difference of the response magnitude or response threshold from that before injection (“Pre”) was plotted and analysed against the time after injection (Fig. 7). The response magnitude of the bafilomycin A1 group tended to decrease after injection of bafilomycin A1, while that of the vehicle group fluctuated, but did not show a steady tendency to decrease (Fig. 7A). The response threshold for the bafilomycin group was higher than that for the vehicle group (Fig. 7B). A two-way ANOVA with repeated measures (see Supplementary Table 3) followed by a post-hoc Bonferroni’s test was performed for the response magnitude and threshold. The response magnitude of the DMSO group did not change significantly with time after the injection (*p *> 0.245). However, in the bafilomycin A1 group, the response magnitude was lower than that of the DMSO group (*p *= 0.038) and lower before the injection at all time points (Fig. 7A, *p* < 0.01 at all time points, Supplementary Table 3). The response magnitude of the bafilomycin A1 group was also significantly lower than that of the DMSO group 40 min after injection (Fig. 7A, *p* < 0.01). In the bafilomycin A1 group, the threshold was significantly higher than that in the DMSO group (*p* = 0.024, main effect of drug, Supplementary Table 3) and also higher 20 min after injection than before (Fig. 7B, *p *= 0.012), while there were no significant changes in the DMSO group (Fig. 7B, *p *= 1). Subsequently, the threshold of the bafilomycin group was significantly higher than that of the DMSO group 20 min after injection (*p* = 0.015). We confirmed that bafilomycin A1 did not affect the response magnitude and response threshold with time in the control fibres obtained from naïve (no RCS) rats (response magnitude: F (2.22, 31.12) = 0.88, *p* = 0.44; threshold: F (2.29, 32.11) = 0.41, *p* = 0.69. n = 15, one-way repeated measures ANOVA).

### Effects of manipulating extracellular matrix proteoglycans on decreased mechanical withdrawal threshold after RCS

Previously, we proposed an extracellular mechanism involving extracellular matrix proteoglycan for the low pH-induced facilitation of mechanical responses based on the inhibitory effects of chondroitin sulfate (CS) and chondroitinase ABC^32^. Therefore, we checked whether extracellular matrix proteoglycan plays a role in behavioural nociceptive hypersensitivity to mechanical stimulation by decreasing pH after RCS. CS (0.01–1 mg/mL) dissolved in tap water was supplied to the rats, allowed to drink freely, and its volume drunk per day was measured. The volume drunk was 93.7 ± 3.8 mL/day/kg body weight for CS 1 mg/mL group, 83.3 ± 7.6 mL/day/kg for CS 0.1 mg/mL group, 93.9 ± 10.1 mL/day/kg for CS 0.01 mg/mL group, 86.5 ± 6.6 mL/day/kg for water group (n = 6 each). There were no significant differences in the volume of drink among the groups with various concentrations of CS (one-way ANOVA, F (3, 20) = 3.042, *p* = 0.0524).

**Figure 8:**
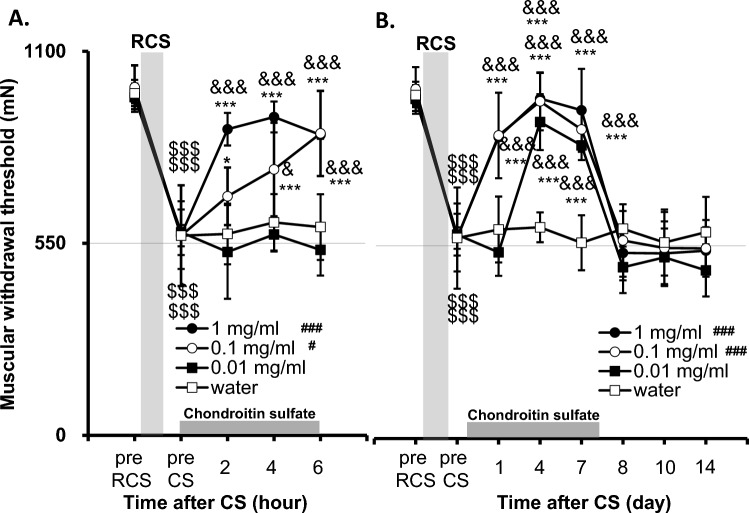
Effects of various doses of CS on the mechanical withdrawal threshold that decreased after RCS. The presentation method was similar to that shown in Fig. 3. (**A**) Time course of the effect of CS over a short period (up to 6 h after starting administration of CS). CS administration was started 1d after RCS. (**B**) Time course of the effect of CS over a longer period (up to 14 days). The gray column above the horizontal axis indicates the period during which CS is supplied. Two-way-repeated measures ANOVA (see Supplementary Table 4) was followed by post-hoc analyses using Bonferroni’s test. #, ###: *p* < 0.05, 0.001 compared with the water group, $$$: *p* < 0.001 compared with “pre RCS”, *, ***: *p* < 0.05,  0.001 compared to “pre CS” (before administration of CS) in each drug group, &, &&&: *p* < 0.05, 0.001 compared with the water group at each time point. Note that groups supplied with CS 0.1 mg/mL and 1 mg/mL showed a significant reversal of the decrease in MMWT from 2 h after the start of CS intake. On the other hand, the group that was supplied CS of the lowest concentration, 0.01 mg/mL showed no reversal of MMWT up to 6 h (**A**), however, this group also showed a significant reversal of MMWT after 4 day-intake of chondroitin sulfate (**B**), that is, the lower the concentration of CS is, the longer the time it takes until analgesic effects appear. When the supply of CS was stopped, the analgesic effect disappeared within a day at all concentrations (**B**).

As seen in Fig. 8A, CS 0.1 mg/mL and 1 mg/mL but not 0.01 mg/mL reversed the decrease in mechanical withdrawal threshold after RCS (*p* < 0.001 in [dose], [time], and [dose] x [time] interactions, two-way repeated measures ANOVA, n = 6 for each dose, see Supplementary Table 4). Compared to the water group, the 0.1 mg/mL and 1 mg/mL groups had significantly higher withdrawal thresholds (*p* = 0.025 and *p* < 0.001 for the 0.1 mg/mL and 1 mg/mL groups, respectively, Bonferroni’s test). A significant change over the water group was observed at 2 h and later in the CS 1 mg/mL group (*p* < 0.001 at all time points), but it was first observed 4 h after the start of CS 0.1 mg/mL intake (*p* = 0.033). A significant reversal of the decreased withdrawal threshold of “pre CS” was observed 2 h and later after starting intake of both CS 0.1 mg/mL and CS 1 mg/mL (*p* = 0.024 [2 h in the CS 0.1 mg/mL group] and* p* < 0.001 [2 h in the CS 1 mg/ml and other time points in both groups], vs. “pre CS”, Bonferroni’s test). Up to 6 h after starting administration, CS 0.01 mg/mL had no effect. Overall dose–response relationship up to 6 h can be seen in AUC graph (Supplementary Fig. S3A). When observed in a longer time range using the data at 6 h as data at 1 day (Fig. 8B), CS 0.1 and 1 mg/mL significantly reversed the decrease in withdrawal threshold after RCS (*p* < 0.001 vs. “pre CS”’, Bonferroni’s test after two-way repeated measures ANOVA, data analyzed between “pre RCS”’ and 8 days, see Supplementary Table 4). CS 0.01 mg/mL also reversed the decreased withdrawal threshold; the reversal was detected 4 days after the start of CS administration, which was the first measurement day in the longer time range (*p *< 0.001 vs. “pre CS”. Bonferroni’s test). The actual reversal may have occurred earlier (2 or 3 days after the start of CS administration). The maximum effect of every dose was similar 4 days after the start of administration of CS. These results showed that a longer time is required with a lower dose until the effect develops (Clear overall dose–response relationship can be seen in Supplementary Fig. S3B in longer time range). However, the analgesic effect of CS disappeared the day after CS administration was stopped (8 days after the start of CS administration), and this quick disappearance was observed irrespective of the dose (Fig. 8B).

**Figure 9:**
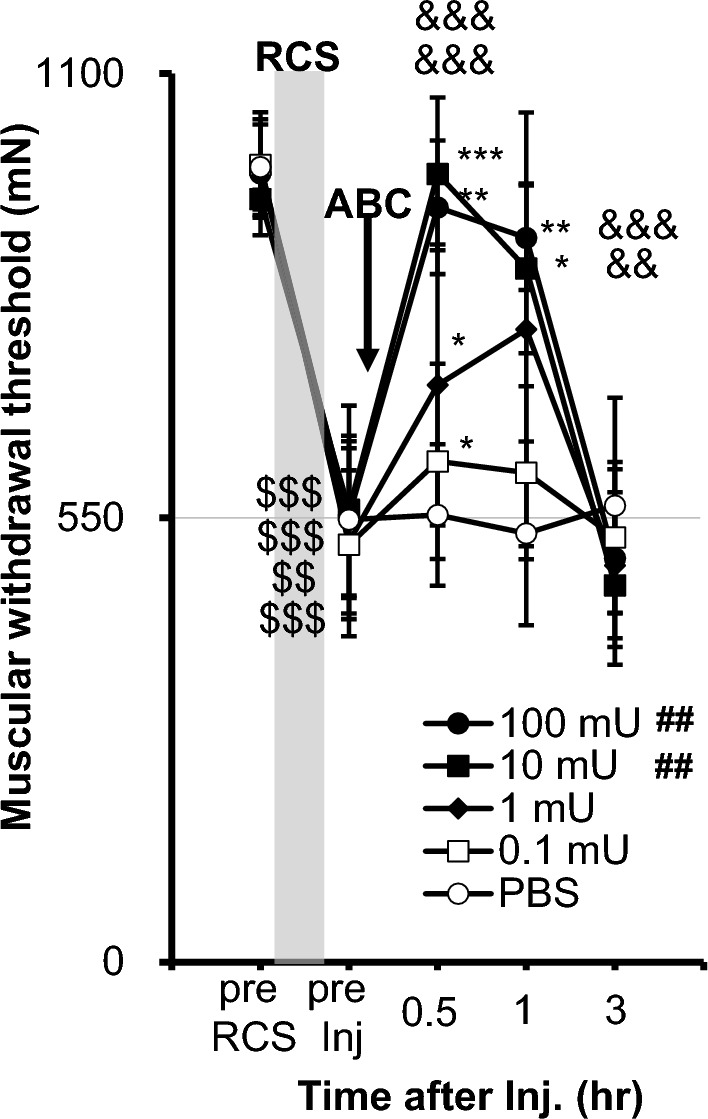
Effects of chondroitinase ABC on the decreased withdrawal threshold after RCS. The presentation method was the same as that shown in Fig. 3. Chondroitinase ABC was administered 3–15 days after RCS. Two-way ANOVA with repeated measures (see Supplementary Table 4) followed by Bonferroni’s post-hoc test. ##*: p* < 0.01 compared to PBS group (main effect of “drug”), $$, $$$: *p* < 0.01,0.001 compared to “pre RCS”, *, **, ***: *p* < 0.05, 0.01, 0.001 compared to “pre Inj” in each drug group, &&, &&&: *p* < 0.01,  0.001 compared to the PBS group at each time point. Note that MMWT was reversed (higher than pre Inj) by chondroitinase ABC at all doses at 0.5 h after injection, and this reversal continued up to 1 h with 10 and 100 mU, and the effects disappeared 3 h after injection. Significantly higher MMWT was observed only in 10 and 100 mU (main effect of drug).

Chondroitinase ABC, which cleaves CS, 10 mU/rat, and 100 mU/rat injected to the right gastrocnemius (GC) muscle, both reversed the decreased mechanical withdrawal threshold after RCS (Fig. 9, p < 0.01 vs. phosphate-buffered saline (PBS, 0.01 M, pH 7.4); two-way repeated measures ANOVA, see Supplementary Table 4). Chondroitinase ABC 10 mU/rat and 100 mU/rat almost completely reversed the decreased withdrawal threshold after RCS 0.5 h and 1 h after injection compared with before injection (“pre Inj”) (*p* = 0.0006–0.0240 and 0.0050–0.0042, respectively, Fig. 9). The withdrawal threshold after 10 mU/rat and 100 mU/rat was significantly higher than PBS 0.5 h (*p* < 0.0001) and 1 h after injection (*p* = 0.0017 and 0.0005, respectively). 1 mU and 0.1 mU injection significantly reversed the decrease in the withdrawal threshold after RCS 0.5 h after injection vs. “pre Inj” (*p* = 0.0385 and *p =* 0.0174, respectively). These effects did not last for long and disappeared 3 h after injection. Overall dose–response can be clearly seen in AUC (the Supplementary Fig. S4).

### Effects of antagonists to acid-sensitive ion channels (ASIC3 and TRPs) on mechanical nociceptive hypersensitivity after RCS and changed expression of these channels in DRGs

**Figure 10:**
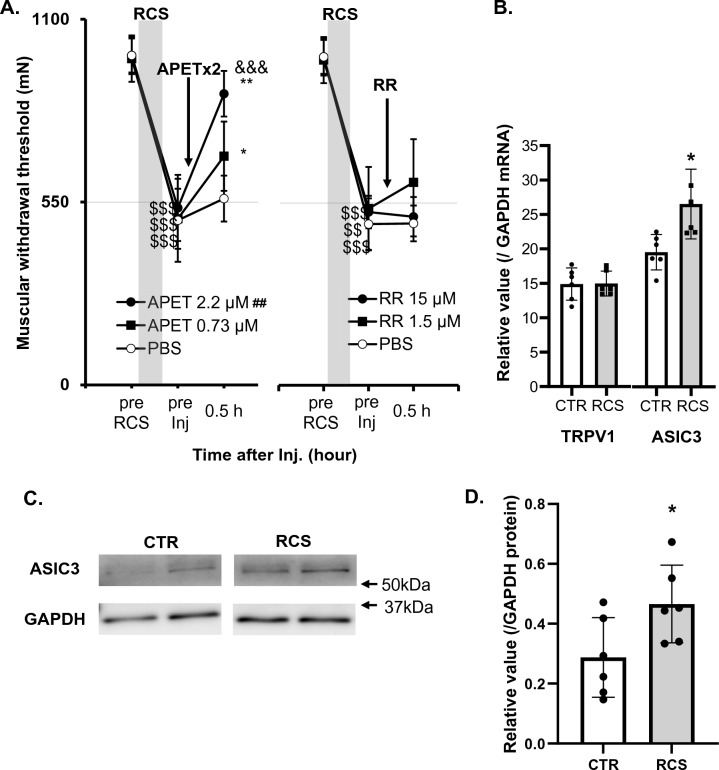
Changes in RCS-induced decrease of MMWT by antagonists to ASIC3 and TRPs (**A**), in mRNAs expression of ASIC3 and TRPV1 (**B**) and in protein of ASIC3 in DRGs after RCS (**C**, **D**). (**A**) The presentation methods are the same as those in Fig. 3. The arrow shows the period at which APETx2 and RR were injected (3–5 d after RCS). Two-way ANOVA with repeated measures (see Supplementary Table 5) followed by Bonferroni’s post hoc test. ##*: p* < 0.01 compared to the PBS group (main effect of the “drug”), $$, $$$: *p* < 0.01, 0.001 compared to “pre RCS”, *, **: *p* < 0.05, 0.01 compared to “pre Inj” in each drug group, &&&: *p* < 0.001 compared to PBS at the time point, APETx2 (APET) 0.73 μM and 2.2 μM reversed the mechanical withdrawal threshold decreased after RCS but RR neither 1.5 μM nor 15 μM did. (**B**) Vertical axis: ASIC3 mRNA and TRPV1 mRNA relative to GAPDH mRNA. DRGs were removed from rats 1 week after RCS. *: *p* < 0.05. Note that only ASIC3 mRNA level increased significantly after RCS. (**C**) Western blot analysis of ASIC3 protein expression in DRGs of RCS and control (CTR) rats. Blots from two rats for each group were represented. GAPDH was used as an internal control. (**D**) ASIC3 protein levels in DRGs quantified from the Western blot (n = 6 for each group). Data were shown as relative values to GAPDH. *: p < 0.05. (See Supplementary Table 1 for statistics summary for (**B**) and (**D**)).

Next, we studied the involvement of acid-sensitive ion channels in the mechanism by which the lowered pH after RCS induces nociceptive hypersensitivity to mechanical stimulation. First, we examined the possible involvement of acid-sensitive ion channels, namely ASIC3 and TRP channels, using APETx2, an ASIC3 antagonist, and ruthenium red (RR), a non-specific TRP antagonist. APETx2 reversed the decrease in withdrawal threshold to mechanical stimulation after RCS (Fig. 10A, p = 0.0139 in [dose], *p* < 0.0001 in [time], *p* = 0.0007 in [dose] × [time] interaction, two-way repeated measures ANOVA, n = 6 each; see Supplementary Table 5). The withdrawal threshold 0.5 h after APETx2 2.2 μM was significantly higher than PBS (*p* < 0.0001), and APETx2 0.73 and 2.2 μM reversed the withdrawal threshold decrease after RCS (*p* = 0.0121 and 0.0022 vs. before injection (“pre Inj”), respectively). However, PBS injection did not change the threshold (*p* = 0.3675) (Fig. 10A, left). Unlike APETx2, the withdrawal threshold after RR was significantly higher than neither after PBS (*p* = 0.2567 for 1.5 μM, *p* > 0.9999 for 15 μM) nor that of before injection (“pre Inj”) after RCS (*p* = 0.8529 for 1.5 μM and *p* = 0.9045 for 15 μM, n = 6) (Fig. 10A, right).

Next, we examined the mRNA expression of TRPV1 and ASIC3 in the DRGs after RCS using reverse-transcription quantitative polymerase chain reaction (RT-qPCR). In accordance with the results of the behavioural experiments described above, ASIC3 mRNA was upregulated (*p* = 0.0150, unpaired t-test, n = 6), but TRPV1 mRNA was not (*p* = 0.953, unpaired t-test, n = 6, Fig. 10B). ASIC3 protein measured by Western blotting was also increased in the DRGs after RCS (*p* = 0.0405, unpaired t-test, Fig. 10C,D).

## Discussion

This study showed that the muscular pH decreased after RCS. The observed pH change was small and not within the acidic range. The pH probe used was large relative to the volume of TA muscle; therefore, it measured the average pH of large muscle areas with tissue fluid. The measured pH change may have been small because of the strong buffering action of the tissue fluid. To measure the pH near the affected muscle fibres’ plasma membrane or near the thin-fibre afferent terminals, the pH must have been much lower, possibly within the acidic range.

Decreases in pH have been reported in muscles during exercise^40,41^, inflammation^13,14^, and ischaemia^42,43^. The mechanism of tissue pH decreases under these conditions has not been fully examined. Herein, a selective inhibitor of V-ATPase, bafilomycin A1, almost completely reversed the lowered muscle pH after RCS indicating that the pH in the muscle of the RCS model was lowered by V-ATPase activation.

Additionally, this study showed that decreased pH in the RCS model is crucial in muscular nociceptive mechanical hypersensitivity after RCS, because subcutaneously injected bafilomycin A1 reversed both muscle pH and mechanical hypersensitivity after RCS. Furthermore, the observation that an inhibitor of H^+^/K^+^ ATPase reversed mechanical hypersensitivity only slightly showed that V-ATPase, but not H^+^/K^+^ ATPase, was mainly involved in reducing muscular pH and the resulting mechanical hypersensitivity.

Carbonic anhydrase (CA) is involved in extracellular pH control in muscles^44^, and its level increases during inflammation^45^. However, its involvement in mechanical allodynia in carrageenan-inflamed muscles has not been identified^46^. A V-ATPase inhibitor, bafilomycin A1, had a clear effect in this study; therefore, we did not examine CA.

Mechanical nociceptive hypersensitivity in the RCS model lasts up to 3 weeks after RCS (Nasu et al*.*^9^ and confirmed in the present observation), and we confirmed that muscle pH was still declining 3 weeks after RCS, though in gastrocnemius muscle (unpublished observation from our lab); however, bafilomycin A1 reversed the decreased MMWT only shortly and partially 3 weeks after RCS, suggesting that in later stages, mechanisms other than activation of V-ATPase play a more important role. The spinal/central mechanism may contribute more to this stage’s behavioural mechanical nociceptive hypersensitivity. We reported the activation of microglia^47^, and its activation was observed from 1 day to 2 weeks after RCS. Based on previous reports, a decrease in 5HT or adrenaline in the brainstem and spinal cord may have changed nociceptive sensitivity^48^. However, this observation was made 1 day after RCS, and neither study has a data on a longer time course^47,48^.

Single fibre recording of C-fibres from the EDL muscle of rats that underwent RCS showed increased mechanical sensitivity (increased response magnitude and reduced response threshold to mechanical muscle stimulation), which confirmed previous results^12^, and this study showed that bafilomycin A1 reversed it. This observation shows that decreased muscular pH is responsible for the increased mechanical sensitivity of the C-fibre muscle afferents, which is the peripheral neuronal mechanism of behavioural nociceptive hypersensitivity to mechanical stimulation in RCS rats.

The mechanism by which V-ATPase is activated in the RCS model will be interesting, although we did not perform any experiments on this point. V-ATPase comprises many subunits, including the V1 domain, which locates in the cytoplasm and hydrolyses ATP, and the V0 transmembrane domain, which transports hydrogen ions and has a binding site for bafilomycin A1^21^. Regulation of V-ATPase activity has been reported to be achieved through the reversible dissociation of V1V0 complexes, control of their cellular localisation (trafficking), and changes in the efficiency of coupling proton transport with ATP hydrolysis^21,49^. Reversible exocytosis and endocytosis of intracellular vesicles containing a high density of V-­ATPases are other mechanisms for controlling V-ATPase activity^50^. Reported factors and conditions that increase the assembly of V-ATPase include glucose starvation in HEK293T cells and LLCPK cells, epidermal growth factor in hepatocytes, amino acid starvation in HEK293T cells, synaptic vesicle recycling in mouse hippocampal neurones, and others^49^. Many changes have been reported to occur in RCS models, such as body weight loss despite increased food intake^51,52^, autonomic dysfunction^53^, increased activity of glia in the spinal dorsal horn^47^, and decrease in 5HT or adrenaline in the spinal cord and brainstem^48^, which would be related to depression^10^ and anxiety-like behaviour^6^. Currently, we have not detected any change in the cellular and membrane expression of V-ATPase by RT-qPCR and western blotting (unpublished observation of our lab). Changes in the body function in RCS listed above are complex compared to studies using cell lines; therefore, it is essential to identify the key factor influencing V-ATPase in the RCS model and then how it influences V-ATPase function could be studied. All of these aspects remain open for future studies.

This study showed that nociceptive mechanical hypersensitivity after RCS was reversed by CS and chondroitinase ABC in a dose-dependent manner. Kubo et al*.*^32^ previously reported that mechanically activated currents were more often facilitated by acid in cultured IB4( +) small DRG neurones than in IB4(-) DRG neurones and that this proton-induced facilitation was suppressed by CS and chondroitinase ABC, but not by inhibitors of broad-spectrum kinases and phospholipase C or by Ca^2+^ depletion from the extracellular solution, indicating no involvement of intracellular signalling pathways. Furthermore, our group showed that a mixture of antagonists against TRPV1 and ASICs reversed proton-induced currents. However, neither TRPVs nor ASICs have been implicated in the proton-induced facilitation of mechanically activated currents, except for rapidly adapting currents. Based on these observations, Kubo et al*.*^32^ proposed a novel extracellular mechanism involving extracellular proteoglycans for proton-induced sensitisation. Our group further showed that this extracellular mechanism works at the tissue level by recording single nerve activities from muscular afferents^33^. Therefore, our present results are consistent with the extracellular mechanism proposed by Kubo et al*.*^32^. One point that differs from their results is that the ASIC3 inhibitor APETx2 reversed behavioural mechanical hypersensitivity after RCS in this study. Kubo’s experiment was performed on cultured DRG neurones obtained from normal rats, whereas the present experiment was performed under chronic conditions after RCS with increased expression of ASIC3 mRNA. The involvement of TRPV1 and other TRP channels in mechanical hypersensitivity was not supported because the non-selective antagonist of TRPs, RR, failed to reverse the decrease in MMWT. Therefore, we consider that ASIC3 was recruited in the proton-induced facilitation of the mechanical response after RCS, in addition to the above-mentioned extracellular mechanism. The involvement of ASIC3 has been reported in mechanical hypersensitivity in iinflammation^28–30^ and nerve injury^31^, and present results further extend its role to another pathological condition, RCS.

In Kubo’s report, mechanically activated currents were mainly sensitised in IB4-positive neurones expressing the extracellular matrix proteoglycan versican^32^. Although our group examined single fibre activity after RCS without identifying IB4 binding properties in this study and a previous study^12^, we found sensitisation to mechanical stimulation in fibres recorded from RCS rats, in which muscle pH was lowered. Because a large percentage of thin-fibre muscle afferents (70.6%) are reportedly IB4-positive^54^, the high percentage of fibres recorded in these studies must be IB4 ( +). If we were to record only IB4( +) fibres, then the mechanical response of the fibres recorded in RCS rats would have been larger than that of the all fibres including IB4(+) and (-) fibres, and the effects of bafilomycin A1 would have been much clearer. Alternatively, CS proteoglycans may exist in tissues in forms other than versican and influence the mechanical sensitivity of thin-fibre afferents.

CS is well known as a slow-acting drug for osteoarthritis and has been officially accepted by the World Health Organization/International League of Associations for Rheumatology Task Force in 1994. It is recommended at doses of approximately 1200–1600 mg/day for oral intake^55^. The dose of CS for the human body is in the range used in this study (approximately 0.9–90 mg/kg [0.01–1 mg/mL × approximately 90 mL/day] in rats vs. 17–22.8 mg/kg in humans). It took a long time (4 days) to develop effects when the lowest dose (0.9 mg/kg (0.01 mg/mL × 90 mL/day) was used in this study, compatible with slow action in clinical usage.

In conclusion, to the best of our knowledge, this study is the first to show that muscle mechanical hypersensitivity after RCS is mediated by lowered muscle pH via V-ATPase. We also showed that low-pH induced muscular mechanical nociceptive hypersensitivity is mediated by extracellular matrix proteoglycans and by upregulated ASIC3. There might be other pathologically painful conditions, without known causes, that are induced by V-ATPase activation. This study will shed light on the mechanism of pain generation under these conditions.

## Materials and methods

### Animals and approval

In this study, we used 237 male Sprague–Dawley rats (SLC Inc., Shizuoka, Japan). We used male rats because muscular mechanical hypersensitivity after RCS was observed in both sexes with only varied durations of hypersensitivity, i.e., 3 weeks in males and 4 weeks in females (data on females are unpublished observations of our lab). For the pH measurement experiments, rats aged 10 weeks were used in the measurement session, and 26 rats were used for the control and 31 for RCS. For the single fibre experiment, rats aged 8–14 weeks (10.8 ± 1.8 weeks, control group, n = 21) and 10–13 weeks old (11.2 ± 1.0 weeks, RCS group, n = 47) were used at the time of the recording. For RT-qPCR and Western blotting, 10-week-old rats (n = 12 each [RT-qPCR] and 6 each [Western blotting] for control and RCS groups) were used. In the MMWT measurement experiments (pharmacology), RCS rats (n = 83) were 9 weeks old at the start of RCS exposure, and control rats (n = 6) were 10 weeks old when measurements were performed (precise allocation of animals were stated in ‘MMWT and pharmacology session’). The rats were housed in plastic cages, containing two or three rats, and had free access to water and rat chow. They were kept in the animal facility at a temperature of 22–24 °C and a 12-h light/dark cycle. All experimental procedures were performed according to the Regulations for Animal Experiments at Chubu University (Approval No. 271008, 2810042, 2910031, 3010072, 202010010, 202010005), the Nihon University School of Dentistry (AP18DEN003), and the Fundamental Guidelines for Proper Conduct of Animal Experiments and Related Activities in Academic Research Institutions in Japan.

### RCS

RCS was applied, as previously described^9^. Figure 1 shows the schedule for RCS exposure. Rats were kept at 4 °C from 19:00 on the day before RCS (day 0) to 10:00 the following morning and then alternately exposed to room temperature (22–24 °C) and cold temperature (4 °C) at 30-min intervals from 10:00 to 17:30. This procedure was repeated for 5 days. After the last exposure to room temperature exposure on the fifth day, the rats were kept in a cold room until 10:00 the next morning (day 6). Two to three rats were housed in each metal-mesh cage, which was fitted with plastic pads (Cozee Pad, 152 mm 89 mm each, Bioresearch Center Inc., Nagoya, Japan) at the bottom to protect rat paws from damage. The rats were exposed to repeated temperature changes in a homemade automated chamber with two separate compartments at different temperatures (one at 22–24 °C and the other at 4 °C). The rats in the cage were automatically transferred from one compartment to another over 30 s using a custom-built device at preset intervals, as described above.

### Measurement of muscle pH

Rats were anaesthetised by inhalation of isoflurane (initially 2%, then kept at 1.3% during the measurement period; FUJIFILM Wako Pure Chemical Corp., Osaka, Japan), and the body temperature was maintained in the physiological range. The pH of the TA muscle was measured using a pH electrode (413 B; Microelectrode Inc., Bedford, NH, USA). The electrode was inserted into the belly of the TA muscle through a guide needle (18 gauge) and stabilised for at least 45 min to minimise pH changes due to tissue damage caused by the insertion of the guide needle and electrode^56^. Rats with bleeding were excluded (n = 1 for the control group and n = 4 for the RCS group). After the measurements the rats were euthanised with CO_2_ (increasing up to 100%).

### Measurement of muscle mechanical withdrawal threshold and pharmacology

To evaluate the magnitude of muscular nociceptive mechanical hypersensitivity, the withdrawal threshold from compression stimulus was measured in awake rats using a Randall–Selitto analgesiometer (Ugo Basile, Comerio, Italy) equipped with a probe of tip diameter 2.6 mm with which the MMWT can be measured^9^. The rats were restrained around the trunk with a towel and treated gently during the experiments. Training sessions were conducted for at least 4 consecutive days. The probe was applied to the belly of the lateral GC muscle through shaved skin. The speed of force applied was set at 157 mN/s, and the cutoff point was set at 2450 mN loading to avoid tissue damage. We have shown in the previous experiment that repeating this measuring procedure for 6 weeks does not produce any change in MMWT in untreated rats^9^.

Each group consisted of six rats, except for bafilomycin A1, a specific inhibitor of V-ATPase, at 4.0 and 13 nmol/kg (n = 5). In the study of dose–response relationship of bafilomycin A1 1 w after RCS, doses of 4.0 and 13 nmol/kg were injected to the same rats five days apart, and doses of 24 nmol/kg and 40 nmol/kg were injected to the different groups of rats. Effects of bafilomycin A1 2 and 3 weeks after RCS were studied on the same rats as that received 40 nmol/kg bafilomycin A1 1 week after RCS. Effects of DMSO at all three time points were studied by injecting to the rats received 40 nmol/kg one day apart. Different doses of PF3716556, an inhibitor of H^+^/K^+^-ATPase, were administered to the same rats one day apart. The CS and chondroitinase ABC series were performed in 24 rats each (n = 6 for each dose). In APETx2 and RR series, rats were divided into two groups and one group received RR 15 μM and APETx2 2.2 μM one day apart. The second group received APETx2 0.73 μM, RR 1.5 μM on the same day because small or no effects were expected.

To make experimenter blinded to the drug and/or dose, and to reduce the effect of injection order, every group was divided into two (three in case of PF3716556), and each subgroup received one drug (dose) or other drug/dose (vehicle was used except bafilomycin A1 4 and 13 pmol/kg, all dose of PF3716556 and APETx2 0.73 μM, RR 1.5 μM series), and this was interchanged on the next injection.

In the proton pump inhibitor series, measurements were performed before RCS exposure; shortly before inhibitor injection; and 1.5, 3 h and 1 d after inhibitor injection 1, 2, and 3 weeks after RCS for the bafilomycin series and 1 week after RCS for the PF3716556 series. In this series, the %MPE of the most effective dose of a drug at the peak time was calculated as follows:

%MPE = 100 × [MMWT at peak time after drug injection − MMWT before injection (pre Inj)]/[MMWT before RCS (pre RCS) − MMWT before injection (pre Inj)].

In experiments with antagonists against ASIC3 (APETx2) and TRPV1 (RR), the withdrawal threshold was measured before RCS, shortly before and 0.5 h after antagonist injection 3–5 days after RCS. In the CS series, measurements were performed before RCS, shortly before CS administration (pre CS) on the first day of CS administration (1 day after RCS), and 2, 4, 6 h (= 1 d), 4, 7, 8, 10, and 14 d after the start of CS administration. In the chondroitinase ABC series, MMWT was measured before RCS and shortly before drug injection (pre Inj) on the day of drug administration (3–15 days after RCS), 0.5, 1, and 3 h after injection.

Area Under the Curve (AUC)s of time-MMWT graphs were calculated using MMWT at ‘pre-Inj’ as the baseline data.

In these experiments, the experimenter was blinded to which animal received which treatment.

### Drugs

Bafilomycin A1 (AdipoGen, Fuellinsdorf, Switzerland), a specific inhibitor of V-ATPase^38^ (4.0–40 nmol/kg, injection volume 250 μL/kg, dissolved and diluted in 100% DMSO[Sigma Aldrich Japan, Tokyo, Japan]), and PF3716556 (TOCRIS Biosciences, Minneapolis, MN, USA), a specific H^+^/K^+^ ATPase inhibitor (0.8–7.6 μmol/kg, injection volume 1500 μL/kg, dissolved and diluted in 100% DMSO), were subcutaneously injected into the posterior region of the neck. The dose was determined according to Hiasa et al.^57^ and Mori et al.^58^. APETx2 (0.73 and 2.2 μM, dissolved in PBS, injection volume 20 μL/rat, Peptide Institute Inc, Ibaraki-shi, Japan), RR (1.5 and 15 μM dissolved in PBS, injection volume 20 μL/rat, Sigma Aldrich) was injected to the right GC muscle. The dose of APETx2 was determined based on Matsubara et al.^59^ and that of RR was based on Fujii et al.^28^. CS (pig origin, 0.01, 0.1, 1.0 mg/mL, Zeria Pharmaceutical Co. Ltd., Tokyo, Japan) was dissolved in drinking water for rats and allowed to drink freely for 7 days starting on the first day after RCS. The ingested volume was measured. Chondroitinase ABC (0.1–100 mU in 20 μL of PBS; Seikagaku Co., Tokyo, Japan) was injected into the right GC muscle. The CS dose was determined based on manufacturer’s data. The Chondroitinase ABC dose was determined based on our previous reports^32,33^.

### Single fibre recording in muscle-nerve preparation ex vivo

Because behavioural muscular mechanical hypersensitivity lasts up to 3 weeks after RCS^9^, recordings were performed in rats within 3 weeks after RCS, the single-fibre recording method was similar to that used in previous experiments^33,60^. The EDL muscle-common peroneal nerve preparation was quickly dissected from the rats sacrificed with CO_2_. The isolated preparation was then placed in the test chamber of an organ bath and maintained at about 34 °C (pH 7.4) with superfusion of warmed modified Krebs–Henseleit solution (Krebs buffer solution), which contained (in mM) 110.9 NaCl, 4.7 KCl, 2.5 CaCl_2_, 1.2 MgSO_4_, 1.2 KH_2_PO_4_, 25.0 NaHCO_3_, and 20.0 glucose, and was continuously bubbled with a 5% CO_2_-95% O_2_ gas mixture. The common peroneal nerve was repeatedly split in the recording chamber of the organ bath filled with paraffin oil until single-fibre activity could be recorded. We recorded only C-fibres (Group IV fibres, conduction velocity < 2.0 m/s), and one fibre was recorded from one preparation. The action potentials were continuously recorded on a computer via an analogue-to-digital converter. These data were analysed on a computer using the DAPSYS data acquisition system (developed by Prof. Brian Turnquist, http://www.dapsys.net/) and Lab Chart Pro Spike Histogram software (ADInstruments Pty Ltd., Bella Vista, Australia). After identifying a single fibre, its receptive field was searched using a glass rod, and the mechanical threshold was quantitatively measured using a servo-controlled mechanical stimulator (manufactured by S. Aizawa, Goto College of Medical Arts and Science,Tokyo, Japan). Mechanical stimuli linearly increasing from 0 to 392 mN over 38 s were applied to the receptive field using a round-tipped probe (ϕ 2.6 mm).

Mechanical stimulation was applied before intramuscular (i.m.) injection of bafilomycin A1 or vehicle, followed by 10, 20, 30, and 40 min after i.m. injection. Bafilomycin A1 was dissolved in DMSO and then diluted with Krebs buffer solution to obtain a 4 μM solution (the final concentration of DMSO was 2.5%). A DMSO solution (2.5%) in Krebs buffer was used to test the effect of the vehicle; 5 μL each was injected with a 30G needle into the muscle near the receptive field.

The fibre was defined as sensitive to the mechanical stimuli when the change in the induced discharge rate met the following criteria, as used in a previous study^60^: (1) net increase in discharge rate > 0.1 Hz during the stimulus period or after 60 s, and (2) instantaneous discharge rate of two consecutive discharges exceeding the mean + 2 standard deviation (SD) of the instantaneous frequencies of the background activity.

The response threshold to the mechanical stimulation was defined as the stimulus intensity when the instantaneous discharge rate exceeded the mean instantaneous background discharge rate for 60 s by 2SD.

### RNA isolation and RT-qPCR

DRGs (L4–6, for TRPV1 and ASIC3) were removed from rats (n = 6) sacrificed by inhalation of CO_2_ gas 1 week after RCS when muscular mechanical hyperalgesia was evident^9^. The excised DRGs were snap-frozen in liquid nitrogen and stored at − 80 °C. Age-matched rats without RCS were used as the controls (n = 6). Total RNAs were extracted using the RNeasy Fibrous Tissue Mini Kit (QIAGEN, Hilden, Germany). Complementary DNAs (cDNAs) were prepared using a high-capacity cDNA reverse transcription kit (Thermo Fisher Scientific Inc., Waltham, MA, USA) with random primers according to the manufacturer’s instructions. The gene-specific primers were designed using NCBI Primer-BLAST (http://www.ncbi.nlm.nih.gov/tools/primer-blast) to be separated by one intron in the corresponding genomic DNA to distinguish them from the genomic amplification. The sequences of the primer pairs were as follows: rat TRPV1, 5’-CCTTCCGCTCTGGCAAGCTGC-3’ and 5’-GTTTACCTCGTCCACCCTGAAACAC-3’; rat ASIC3, 5’-TCTGGCAACACTCTGCTCCAGG-3’ and 5’-TTGGTGACAGCACAGGGAGTGGT-3’; rat glyceraldehyde-3-phosphate dehydrogenase (GAPDH), 5’-GCCTGGAGAAACCTGCCAAG-3’ and 5’-CCTCAGTGTAGCCCAGGATG-3’. q-PCR was performed with PowerUp™ SYBR™ Green Master Mix and 7500 Real-Time PCR system (Applied Biosystems™, Thermo Fisher Scientific Inc., Waltham, MA, USA) using a two-step cycling of 40 cycles at 95 °C for 15 s for denaturation, followed by extension at 60 °C for 60 s. For relative quantification, the samples were run with standards made by fourfold serial dilutions of whole mixtures of cDNAs of rat DRGs. All samples and standards were run in duplicates and averaged. The relative mRNA levels of the target genes, obtained using the standard curve method, were normalised to the levels of GAPDH mRNA as an internal control in each sample. The specificity of the PCR amplification was confirmed using melting curve analysis.

### Western blotting

SD rats 1 week after RCS (n = 6) and without RCS (control, n = 6) were sacrificed by inhalation of CO2 and rapidly exsanguinated by transcardiac perfusion with 500 mL ice-cold PBS. After spinal column was opened, bilateral DRGs (L4–6) were removed and stored at -80 °C. DRGs were homogenized in ice-cold lysis buffer containing 1% Nonidet P-40, 25 mM Tris–HCl (pH7.5), 10 mM EDTA, 10 mM EGTA supplemented with Protease Inhibitor Cocktail (Roche Diagnostics, Mannheim, Germany), 2 mM PMSF, 10 mM Na_3_VO_4_, and 100 mM NaF, followed by sonication. Tissue homogenates were centrifuged at 12,000 × *g* for 15 min at 4 °C and supernatant was used for analysis. Protein concentration of the tissue lysate was measured by BCA Protein Assay Kit (Takara Bio Inc. Kusatsu, Japan). The lysate was mixed in SDS sample buffer (at final, 2% SDS, 62.5 mM Tris–HCl (pH 6.8), 10% glycerol and 10% 2-mercaptethanol) and boiled for 5 min at 97 °C. The sample solution containing 5 µg protein were separated by SDS-PAGE using 5–20% gradient gel (e-PAGEL, ATTO Corp., Tokyo, Japan) and electroblotted onto PVDF membranes (Immobilon-P, Millipore, Bedford, MA, USA). The membrane was cut into upper and lower pieces at the level of the 50 kDa size marker, and then soaked in a blocking solution containing 5% normal goat serum in Blocking One (Nakarai Tesque Inc., Kyoto, Japan) for 30 min at RT. The upper piece of membrane was probed with anti-ASIC3 antibody raised in rabbit (1:200, GeneTex, Irvine, CA, USA) and goat anti-rabbit IgG conjugated with HRP (1:2000, Sigma Aldrich), and the lower piece with mouse anti-GAPDH antibody (1:10,000, GeneTex) and goat anti-mouse IgG conjugated with HRP (1:1000, Thermo Fisher Scientific Inc.) to use as loading control. Incubation with the primary antibodies was carried out overnight at 4 °C, and with the secondary antibodies for 30 min at RT, in the blocking solution. Immunostaining was visualised by ECL Prime (GE Healthcare, Buckinghamshire, UK) and chemiluminescence was detected by ImageQuant LAS-4000 (GE Healthcare). Quantification of the western blot was carried out using Image J.

### Statistical analysis

Results of single fibre recording were expressed as median and interquartile range (IQR) except the threshold of the baseline mechanical response from controls and RCS rats and mechanical response magnitude before drug application, which were expressed as the mean and standard deviation (SD). The former was analysed with Mann–Whitney U test, and the latter, pH data (expressed as mean and SD), and data of mRNA and protein were analysed using unpaired t-test with /without Welch’s correction. Results of normality test and equal variance test, and analysis methods are summarized in Supplementary Table 1.

The effects of DMSO and bafilomycin injection on single fibre activity were analysed using two-way ANOVA with repeated measures followed by Bonferroni’s test unless otherwise stated. Behavioural experiments measuring MMWT were analysed using two-way ANOVA with repeated measures, followed by post hoc analysis with Bonferroni’s test. When normality (Shapiro–Wilk test) and/or equal variance (Brown–Forsythe test) were not assumed, the degree of freedom was adjusted with Geisser-Greenhouse’s epsilon, and post-hoc analysis was performed using Bonferroni’s test. A comparison of %MPE between bafilomycin A1 and PF3716556 was performed using an unpaired t-test with Welch’s correction. The volume of CS solution consumed by rats was analysed using one-way ANOVA. Results of normality test, equal variance test, and F values, p values of all ANOVA are listed in Supplementary Tables 2–5.

Statistical analysis was performed using the Sigma plot ver. 13 and Prism ver. 9, except for the results of the single fibre recording, which were analysed using SPSS Ver. 25. The significance level was set at *p* < 0.05.

### Supplementary Information

Supplementary Legends.

Supplementary Information.

## Data Availability

Data supporting the present findings are available from the corresponding author upon reasonable request.
