# Supplementary material for: Vacuolar-ATPase-mediated muscle acidification caused muscular mechanical nociceptive hypersensitivity after chronic stress in rats, which involved extracellular matrix proteoglycan and ASIC3
Source: Sci Rep. 2023 Aug 21;13:13585. doi: 10.1038/s41598-023-39633-1 (PMC10442418; doi:10.1038/s41598-023-39633-1)
Supplement: Supplementary file 1 — Supplementary Legends. [file 41598_2023_39633_MOESM1_ESM.docx]

**Legend for Supplementary Tables**

Supplementary Table 1. Summary of statistics for comparison between two pairs

Supplementary Table 2. Summary of statistics for the effects of bafilomycin A1 on the muscular mechanical withdrawal threshold (MMWT) after RCS.

Statistical analyses using two-way repeated measures ANOVA are listed in the table (same in the tables 3-5).

Supplementary Table 3. Summary of statistics for the effect of PF3716556 on the MMWT after RCS and the effects of bafilomycin A1 on the muscle thin-fibre responses to mechanical stimuli in single fibre recordings.

Supplementary Table 4. Summary of statistics for the effects of chondroitin sulfate and chondroitinase ABC on MMWT.

Supplementary Table 5. Summary of statistics for the effects of a specific ASIC3 antagonist APETx2 and a non-specific TRP antagonist RR on MMWT.

Legend for Supplementary Figures

Fig. S1. AUC of dose-response to bafilomycin A1 on decreased MMWT after RCS, based on Fig. 3.

A: Result obtained 1 week after RCS. Statistical analysis was performed with one way ANOVA with Welch’s correction, followed by Dunnett test. AUC was significantly greater than DMSO at 40 nmol/kg (*** p < 0.001).

B: Result obtained 2 weeks after RCS. Unpaired t-test with Welch’s correction. AUC was significantly greater than DMSO at 40 nmol/kg (* p < 0.05).

C: Result obtained 3 weeks after RCS. Unpaired t-test with Welch’s correction. No significant effect was found. D: Result obtained from normal animals (without RCS).

Fig. S2. AUC of dose-response to PF3716556.

Data presented in Fig. 4 was used. Statistical analysis was performed with one way ANOVA with Welch’s correction, followed by Dunnett test. AUC was significantly larger than DMSO only at 2.5 μmol/kg (* *p* < 0.05).

Fig. S3. AUC of dose- response to chondroitin sulfate.

Data presented in Fig. 8 was used. Statistical analysis was performed with one way ANOVA with Welch’s correction, followed by Dunnett test. *, **, *** p < 0.05, p < 0.01, p < 0.001 compared to water. A: Short time effect, B: Long time effect. Note that AUCs of both 0.1 and 1 mg/ml were significantly greater than that of water in short time (in A), but additionally AUC of 0.01 mg/ml was significantly greater than that of water in longer time (in B).

Fig. S4. AUC of dose-response to chondrotinase ABC

Data presented in Fig. 9 was used. Statistical analysis was performed with one way ANOVA with Welch’s correction, followed by Dunnett test. AUCs of doses of 10 and 100 mU were greater than that of PBS (* *p* < 0.05 compared to PBS).
