# Supplementary material for: Vacuolar-ATPase-mediated muscle acidification caused muscular mechanical nociceptive hypersensitivity after chronic stress in rats, which involved extracellular matrix proteoglycan and ASIC3
Source: Sci Rep. 2023 Aug 21;13:13585. doi: 10.1038/s41598-023-39633-1 (PMC10442418; doi:10.1038/s41598-023-39633-1)

Supplementary information

Vacuolar-ATPase-mediated muscle acidification caused muscular mechanical nociceptive hypersensitivity after chronic stress in rats, which involved extracellular matrix proteoglycan and ASIC3

Teruaki Nasu, Amane Hori, Norio Hotta, Chiaki Kihara, Asako Kubo, Kimiaki Katanosaka, Masamitsu Suzuki, Kazue Mizumura*

Supplementary Table 1

Comparison between two groups

Treatment, measurementt	Figure No.	Normality test	Equal variance	analysis method	n	p values
pH of the muscle (control vs RCS)	Fig. 2	P = 0.2168, 0.3277 (normality assumed, Shapiro-Wilk test)	P= 0.0314 (equal variance not assumed, F test)	unpaired t-test with Welch's correction	n=12 each	P= 0.0245
Comparison of %MPE of bafilomycin A1 40 nmol/kg and PF3716556 2.5 µmol/kg	In the text	P < 0.706, p < 0.8799 (normality assumed, Shapiro Wilk test)	p = 0.0176 (Equal variance not assumed)	Unpaired t-test with Welch's correction	n = 6 each	p = 0.0017
pH of the muscle (control animal, DMSO or BafilomycinA1 inj)	Fig. 5A	P = 0.7204, 0.1073 (normality assumed, Shapiro-Wilk test)	P = 0.1051 (equal variance assumed, F test)	Unpaired t-test with Welch's correction	n=7 each	P = 0.9461
pH of the muscle (RCS animal, DMSO or BafilomycinA1 inj)	Fig. 5B	P = 0.9412, 0.1243 (normality assumed, Shapiro-Wilk test)	P= 0.8582 (equal variance assumed, F test)	Unpaired t-test with Welch's correction	n = 10 (DMSO) n = 9 (bafilomycin A1)	P = 0.0244
Basic characteristics of recorded fibers (Conduction velocity)	In the text	P = 0.087, <0.001 (normality not assumed, Shapiro-Wilk test)	P = 0.269 (equal variance assumed, Levene's test)	Mann-Whitney U test	n = 21 (control) n = 47 (RCS)	P = 0.094
Basic characteristics of recorded afferents (background discharges)	In the text	P <0.001, <0.001 (normality not assumed, Shapiro-Wilk test)	P = 0.951 (equal variance assumed, Levene's test)	Mann-Whitney U test	n = 21 (control) n = 47 (RCS)	P = 0.746
Baseline mechanical response of thin-fibres (response magnitude)	Fig. 6C upper graph	P = 0.005, 0.189 (normality not assumed, Shapiro-Wilk test)	P = 0.519 (equal variance assumed, Levene's test)	Mann-Whitney U test	n = 21 (control) n = 47 (RCS)	P = 0.015
Baseline mechanical response of thin-fibres (response threshold)	Fig. 6C lower graph	P = 0.00025, <.00001 (normality not assumed, Shapiro-Wilk test)	P = 0.011 (equal variance not assumed, Levene's test)	Unpaired t-test with Welch's correction	n = 21 (control) n = 47 (RCS)	P = 0.034

Supplementary Table 1 (continued)

Comparison between two groups

Treatment, measurementt	Figure No.	Normality test	Equal variance	analysis method	n	p values
Mechanical response magnitude of RCS fibres before drug application	In the text	P = 0.311, 0.956 (normality assumed, Shapiro-Wilk test	P=0.116 (equal variance assumed, Levene's test)	Unpaired Student t-test	n = 26 (bafilomycin) n = 12 (DMSO)	p = 0.257
Mechanical response threshold of RCS fibres before drug application	In the text	P <.001, <.001 (normality not assumed, Shapiro Wilk test)	P = 0.248 (equal variance assumed, Levene's test)	Mann-Whitney U test	n = 26 (bafilomycin) n = 12 (DMSO)	P = 0.376
TRPV1 mRNA	Fig. 10B	P = 0.551(normality assumed, Shapiro-Wilk test)	P = 0.775 (equal variance assumed, Brown-Forsythe test)	Unpaired Student t-test	n = 6 each	P = 0.953
ASIC3 mRNA	Fig. 10B	P = 0.136 (normality assumed, Shapiro-Wilk test)	P = 0.290 (equal variance assumed, Brown-Forsythe test)	Unpaired Student t-test	n = 6 each	P =0.0150
ASIC3 protein	Fig. 10D	P < 0.4578, <0.5089 (normality assumed, Shapiro Wilk test)	P = 0.9563 (equal variance assumed, F test)	Unpaired Student t-test	n= 6 each	P = 0.0405

Supplementary Table 2.

Treatment	Figure No.		analysis method	factors	F values	p values	Note (Shapiro-Wilk test was used for normality test, and Brown–Forsythe test was used for equal variance test
Effect of bafilomycin A1 on MMWT	Fig 3A	1 w aft RCS	2-way ANOVA with repeated measures				normality test p = 0.730, equal variance test p = 0.095
				time	F (4, 92) = 168,481	P < 0.001	
				drug	F (4, 23) = 6.703	P < 0.001	
				time x drug interaction	F (16, 92) = 8,047	P < 0.001	
	Fig. 3B	2 w aft RCS	same as above				sphericity not assumed, degree of freedom was adjusted with Geisser-Greenhouse's epsilon.
				time	F (2.76601574, 27.6601574) = 53.0221	P < 0.0001	
				drug	F (1, 10) = 24.1450923	P = 0.0006	
				time x drug interaction	F (4, 40) = 9.65186043	P < 0.0001	
	Fig. 3C	3 w aft RCS	same as above				normality test p=0.808, equal variance test p = 0.997
				time	F (4, 40) = 68.506	P < 0.001	
				drug	F (1, 10) = 1.061	P = 0.327	
				time x drug interaction	F (4, 40) = 4.056	P = 0.007	
	Fig 3D	Control (no RCS)	same as above				normality test p =0.387, equal variance test p = 0.409
				time	F (3,30) = 0.437	P = 0.728	
				drug	F (1, 10) = 0.0111	P = 0.918	
				time x drug interaction	F (3, 30) = 0.460	P = 0.712	

Supplementary Table 3

Treatment	Figure No.		analysis method	factors	F values	p values	note
Effect of PF	Fig. 4		2-way ANOVA with repeated measures				normality test p = 0.739,<br>equal variance test p = 0.666
				time	F (4,80) = 303.858	P < 0.001	
				drug	F (3, 20) = 1.574	P = 0.227	
				time x drug interaction	F (12, 80) = 2.751	P = 0.004	
Single fiber recording	Fig.7A	response magnitude	same as above				sphericity assumed
				time	F (4,144) = 8.44	P < 0.001	
				drug	F (1,36) = 4.66	P = 0.038	
				time x drug interaction	F (4,144) = 3.09	P = 0.018	
	Fig 7B	response threshold	same as above				sphericity not assumed, degree of freedom was adjusted with Geisser-Greenhouse's epsilon.
				time	F(2.84, 102.15) = 0.88	P =0.451	
				drug	F(1, 36) = 5.58	P=0.024	
				time x drug interaction	F(2.84,102.15) = 3.04	P=0.035	

Supplementary Table 4

Treatment	Figure No.		analysis method	factors	F values	p values	note
Effects of chondroitin sulfate	Fig. 8A	up to 6 hs	2-way ANOVA with repeated measures				Normality Test p = 0.519, Equal Variance Test p = 0.124
				time	F(4,80) = 137.522	p < 0.001	
				drug	F(3,20) = 14.223	P < 0.001	
				time x drug interaction	F(12,80) = 12.91	p < 0.001	
	Fig.8B	longer time	same as above				Normality Test p = 0.753 Equal Variance Test p = 0.297
				time	F(5,100) = 165.502	p < 0.001	
				drug	F(3, 20) = 11.586	p < 0.001	
				time x drug interaction	F(15,100) = 16.691	p < 0.001	
Effects of chondroitin ase ABC	Fig 9.		same as above				sphericity not assumed, degree of freedom was adjusted with Geisser-Greenhouse's epsilon.
				time	F (2.28928329, 57.2320821) = 95.4719715	P <0.0001	
				drug	F (4, 25) = 6.89310696	P =0.0007	
				time x drug interaction	F (16, 100) = 5.87206422	P <0.0001	

Supplementary Table 5

Treatment	Figure No.		analysis method	factors	F values	p values	note
Effect of APETx2 on MMWT	Fig 10A, left (APETx2)		2-way ANOVA with repeated measures				sphericity not assumed, degree of freedom was adjusted with Geisser-Greenhouse's epsilon.
				time	F (1.87284114, 26.2197760) = 144.715122	P <0.0001	
				drug	F (2, 14) = 5.89997609	P =0.0139	
				time x drug interaction	F (4, 28) = 6.63304939	P =0.0007	
Effect of ruthenium red on MMWT	Fig 10A, right (RR)		same as above				same as above
				time	F (1.96942453, 27.5719435) = 204.885677	P <0.0001	
				drug	F (2, 14) = 1.23684694	P =0.3202	
				time x drug interaction	F (4, 28) = 1.27967896	P =0.3016	

Suppl. Fig.S1

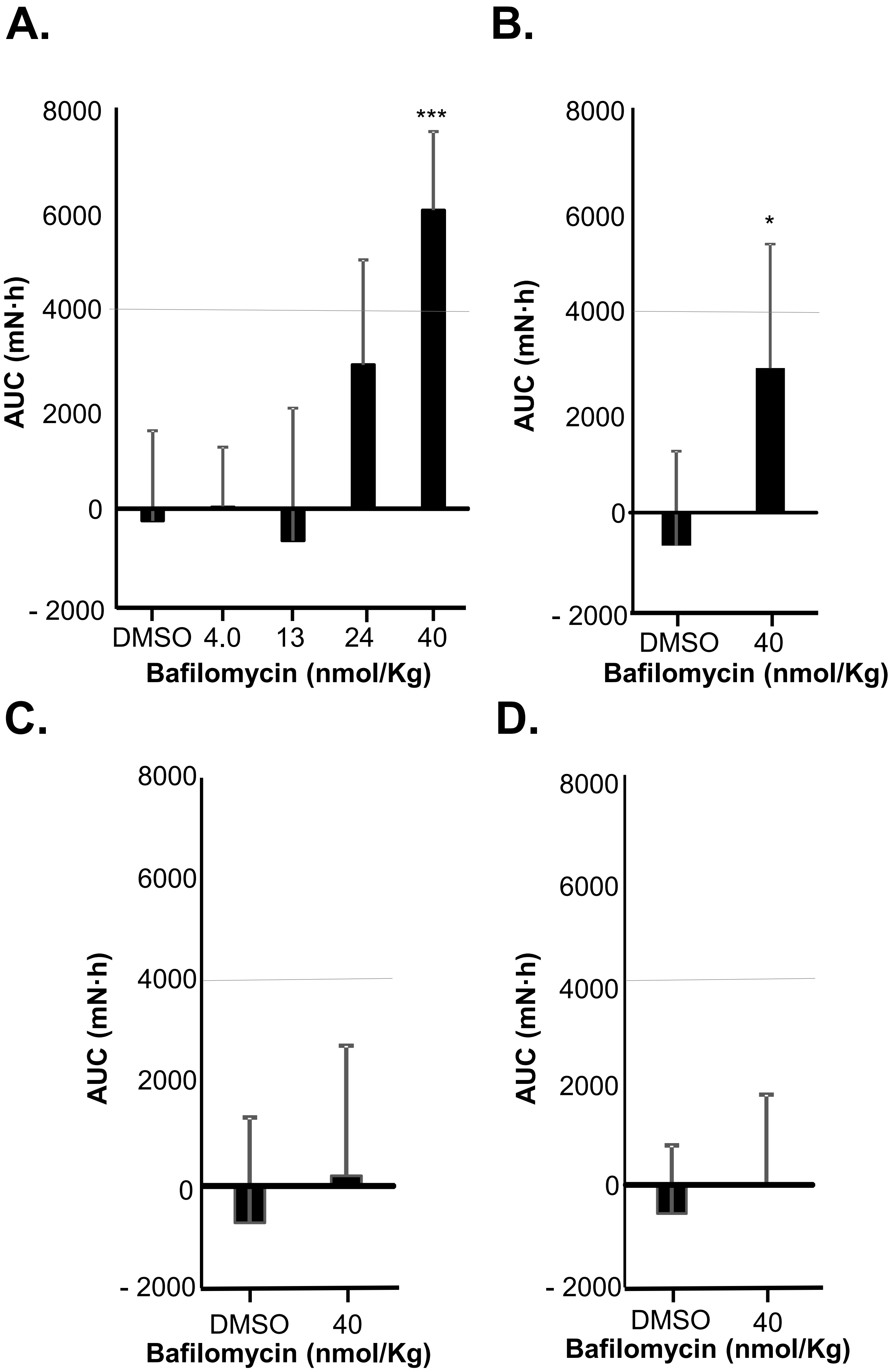
A.
B.
C.
D.
8000
6000
4000
2000
0
- 2000
AUC (mN·h)
***
*
DMSO
4.0
13
24
40
Bafilomycin (nmol/Kg)

Suppl. Fig. S2

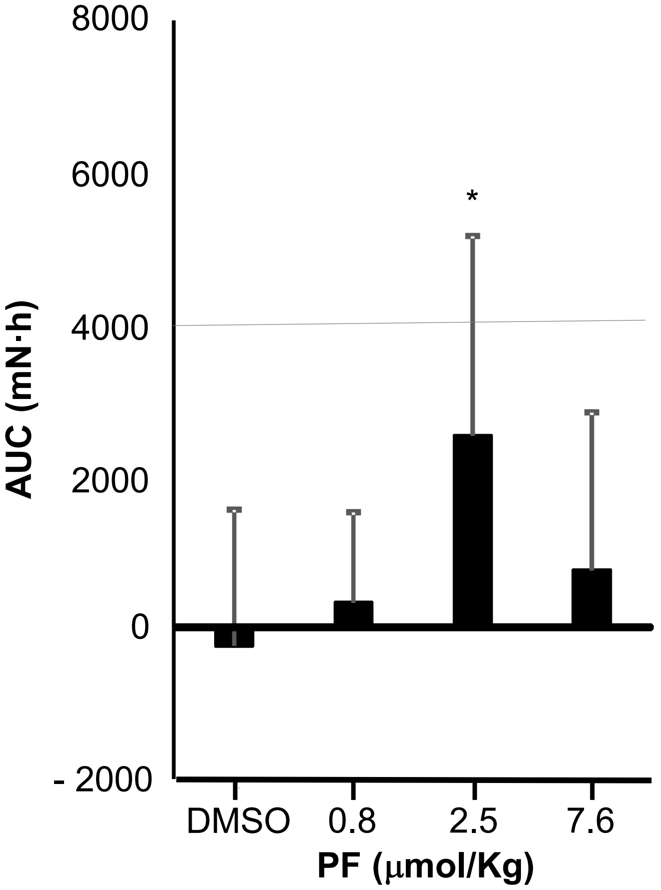

Suppl. Fig. S3.

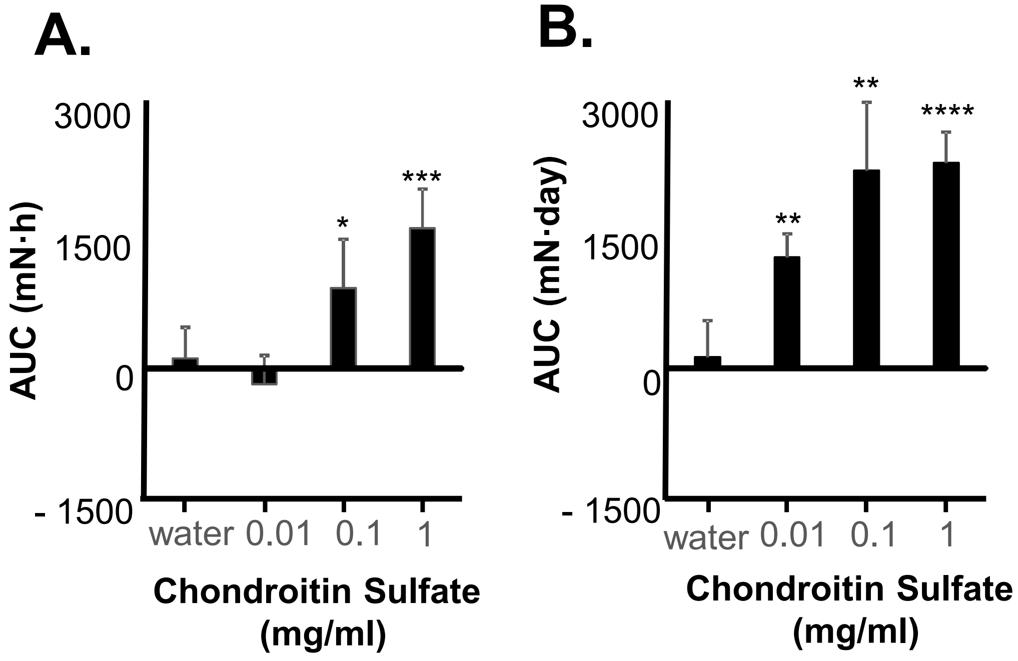
A.
3000
1500
0
- 1500
AUC (mN·h)
*
***
water 0.01 0.1 1
Chondroitin Sulfate
(mg/ml)
B.
3000
1500
0
- 1500
AUC (mN·day)
**
**
****
water 0.01 0.1 1
Chondroitin Sulfate
(mg/ml)

Suppl. Fig.S4

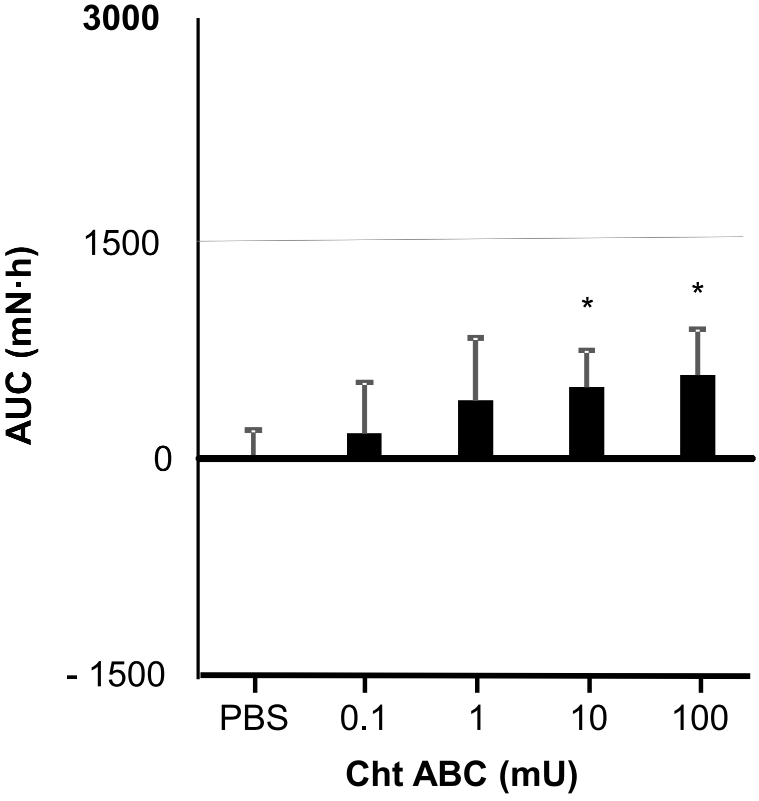

Supplement: Supplementary file 2 — Supplementary Information. [file 41598_2023_39633_MOESM2_ESM.pdf]
